# A strain-programmed lignin-based Janus patch for rapid healing of postoperative rectal wounds

**DOI:** 10.7150/thno.115444

**Published:** 2025-06-20

**Authors:** Yi Yu, Jiaxin Pan, Jianbo Zhang, Zhengdong Zhang, Chenghao Qiu, Yiming Xiang, Yizhou Zhu, Zhanjun Wu, Peng Yu, Tong Li, Lei Tan

**Affiliations:** 1 Department of Orthopaedics, Union Hospital, Tongji Medical College, Huazhong University of Science and Technology, Wuhan 430022, Hubei, China.; 2 School of Fiber Engineering and Equipment Technology, Jiangnan University, Wuxi 214401, China.; 3 Department of Gastrointestinal Surgery, the Second Affiliated Hospital of Chongqing Medical University, 74 Linjiang Road, Yuzhong District, Chongqing 400010, China.; 4 College of Biomedical Engineering, South-Central Minzu University, Wuhan 430074, China.; 5 School of Materials Science and Engineering, Wuhan Institute of Technology, Wuhan 430205, China.; 6 Department of Orthopaedics and Traumatology, Li Ka Shing Faculty of Medicine, The University of Hong Kong, Pokfulam, Hong Kong 999077, China.

**Keywords:** strain-programmed patch, wound healing, antibacterial, antioxidant, bioadhesive

## Abstract

**Rationale:** Clinically, patients experience severe pain, frequent bleeding, and delayed wound healing after hemorrhoidectomy due to recurrent fecal contamination during postoperative dressing changes and bowel movements, which exacerbate wound irritation.

**Methods**: In this study, we developed a strain-programmed lignin-based multifunctional Janus patch (S-SFR@AGL) to accelerate rectal wound repair. This patch features a pre-stretched fluorinated silicone rubber side with robust anti-biofouling and strain-programmed properties, paired with a lignin-based hydrogel side offering potent antibacterial, antioxidant, bioadhesive, and hemostatic capabilities.

**Results**: By modulating the wound's mechanical microenvironment and combating infection-induced inflammation, the patch healed infected rat rectal wounds within one week via effective bacterial clearance, attenuated inflammatory responses, and promoted muscle contraction and epithelial cell differentiation.

**Conclusions**: This Janus patch could shorten intestinal wound healing time, potentially optimizing postoperative management after intestinal lesion resection.

## Introduction

Hemorrhoids, a prevalent anorectal disorder, result from engorgement or congestion of the venous plexus in the anal canal or lower rectum, leading to vascular swelling 1. Epidemiological studies indicate a prevalence of up to 40% among adults in Western nations 2. As the Chinese proverb notes, "Nine out of ten people have hemorrhoids," underscoring its high incidence—particularly among office workers with prolonged sitting, pregnant women, and individuals with constipation or gastrointestinal disorders. Patients typically present with symptoms including rectal bleeding, anal pruritus, pain, and tissue prolapse during defecation 3. Mild cases can be managed conservatively through dietary modifications (e.g., increased fluid/fiber intake), medication, and avoidance of prolonged straining. Severe cases often require surgical intervention. Postoperatively, wounds are intentionally left unsutured to facilitate natural healing via secondary intention. To control bleeding and prevent infection, patients undergo frequent dressing changes and sitz baths 4, 5. Due to the open wounds' rich innervation, defecation and dressing changes frequently cause significant bleeding and sharp pain. Concurrently, patients must perform painful anal dilation exercises to prevent stenosis 6. Collectively, current post-hemorrhoidectomy management remains suboptimal. Given the substantial postoperative discomfort, alongside risks of infection, inflammation, and hemorrhage, there is an urgent clinical need for a specialized rectal wound adhesive patch to optimize postoperative recovery.

For general skin wounds, diverse clinical dressings function as temporary physical barriers to isolate wounds from external contaminants, accelerating healing and tissue regeneration. However, these conventional dressings are often unsuitable for post-hemorrhoidectomy wounds. Hydrogels, with their exceptional biocompatibility, bioadhesion, tunable physico-mechanical properties, and capacity for bioactive compound integration 7-10, show promise for repairing moist mucosal wounds. Previous studies have developed tissue-adhesive patches that bond to wet tissues via hydrogen/amide bonds, demonstrating efficacy in organ hemostasis and infected wound management 11-16. Nevertheless, in the complex rectal wound environment—characterized by peristalsis-induced mechanical stress and persistent exposure to fecal contaminants and bacteria—conventional hydrogel patches face clinically relevant limitations: (i) Lack of anti-fouling functionality, permitting persistent surface accumulation of feces and bacteria; (ii) Non-specific adhesion to wounds and adjacent healthy tissues, risking rectal adhesions; (iii) Insufficient mechanical robustness to withstand peristaltic motion, causing patch dislodgement.

**Figure 1:**
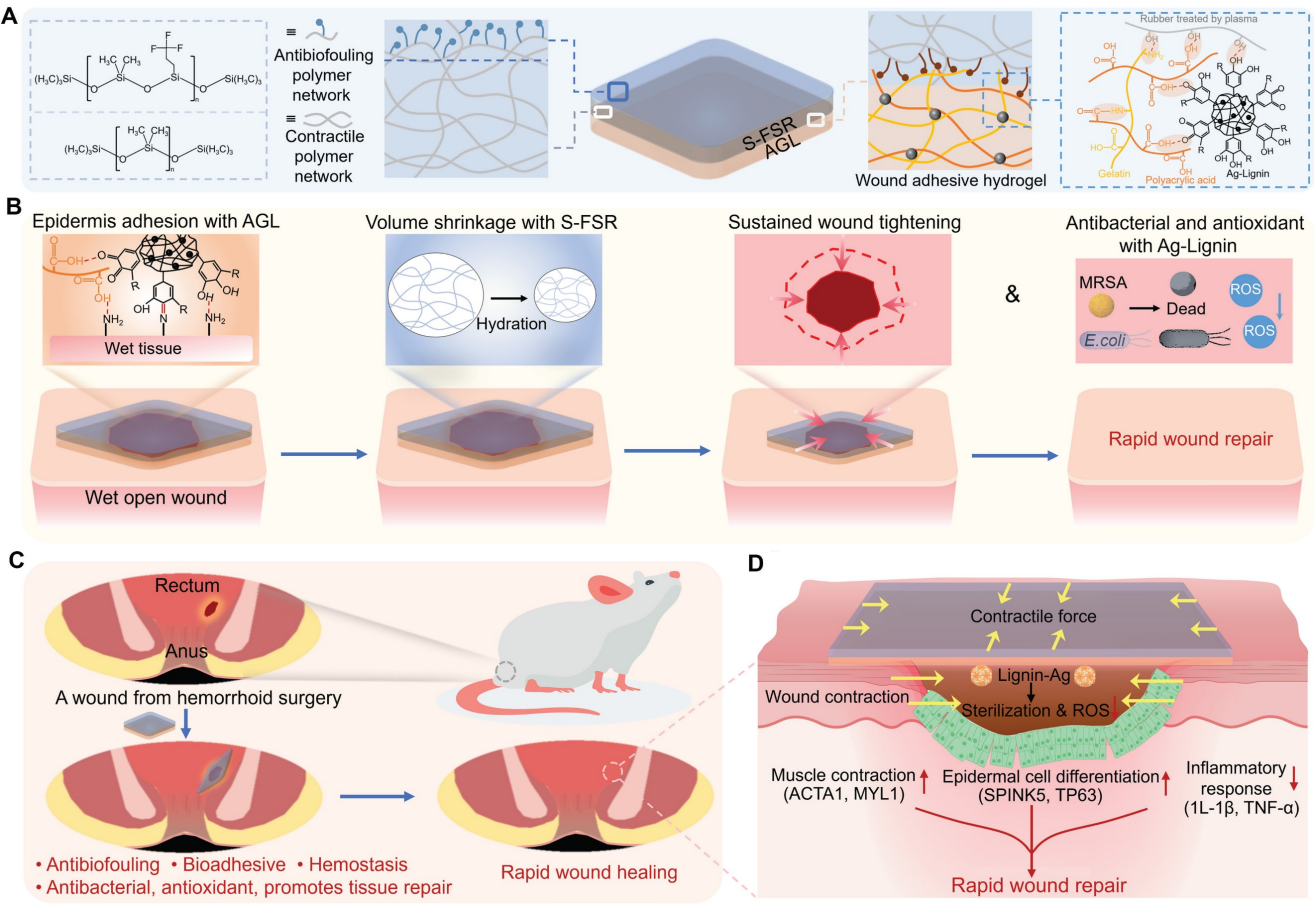
** A strain-programmed lignin-based Janus patch for rapid healing of rectal postoperative wounds.** A. Multifunctional diagram of the patch. B. Schematic illustration of programmed strain release by the patch for moist wound applications. C. Validation of the rat model of post-hemorrhoidectomy wound healing. D. Therapeutic efficacy of the S-SFR@AGL patch.

Hence, to address the complex rectal wound environment, an ideal patch should: (i) Achieve robust adhesion to moist tissue for complete wound coverage; (ii) Prevent surface fouling by fecal contaminants; (iii) Simultaneously inhibit infection, inflammation, and bleeding; (iv) Accelerate wound healing through microenvironment modulation. In this work, we designed a strain-programmed multifunctional lignin-based Janus patch comprising: (i) a pre-stretched fluorinated silicone rubber side that confers anti-biofouling capability and programmable strain retention (mechanism: pre-applied strain stores mechanical stress for controlled release under physiological conditions); (ii) a silver-lignin hydrogel side delivering potent antibacterial, antioxidant, bioadhesive, and hemostatic properties (Figure 1A). Upon application to moist wound tissue, the patch establishes secure bioadhesion and delivers a uniform contractile force to modulate the wound's mechanical microenvironment, thereby actively promoting tissue contraction (Figure 1B). The effectiveness of this patch was validated in a rat model of post-hemorrhoidectomy wound healing, where it demonstrated rapid wound repair by preventing infection, enhancing muscle contraction and epithelial cell differentiation, and suppressing inflammatory responses (Figure 1C-D).

## Materials and Methods

### Chemical reagents and materials

Acrylic acid, gelatin, Ag(NO)_3_, potassium persulfate, 2,2'-Azino-bis (3-ethylbenzothiazoline-6-sulfonic acid) diammonium salt (ABTS), 2,2-Diphenyl-1-picrylhydrazyl were purchased from Shanghai Macklin Biochemical Co., Ltd. Sodium lignosulfonate, trimethoxy (3, 3, 3-trifluoropropyl) silane, glutaric dialdehyde solution, ammonium hydroxide solution were purchased from Shanghai Aladdin Bio-Chem Technology Co., Ltd. Ethanol anhydrous were purchased from Sinopharm Chemical Reagent Co., Ltd. Component A (Silicone dioxide/35%, Vinyl silicone/47%, Methyl silicone oil/17.8%, Platinum water/0.2%) and Component B (Silicone dioxide/35%, Vinyl silicone oil/47%, Methyl silicone oil/10.2%, Hydrogen containing silicone oil/7.8%) were purchased from Hui Zhou Hongyejie Technology Co., Ltd.

### Preparation of modified silicone rubber (SR)

Mixed components A and B in a mass ratio of 1:1 and allowed the mixture to cure at room temperature. When the silicone rubber reached the semi-cured stage, a fluorination treatment was performed on one side by depositing trimethoxy (3,3,3-trifluoropropylmethyl) silane onto its surface. Once the silicone rubber had fully cured, the other side was plasma treated to graft hydroxyl groups onto it (FSR). Finally, the modified silicone rubber was uniformly stretched in all four directions and secured in place (S-FSR).

### Preparation of lignin-Ag

0.1 g of sodium lignosulfonate was dissolved in 5 mL of deionized water, and the mixture was uniformly stirred for 10 min. To 7.35 mL of silver nitrate solution, ammonia was added dropwise, resulting in the formation of a light yellow precipitate. The addition of ammonia continued until the precipitate dissolved, yielding a silver-ammonia solution. This silver-ammonia solution was then added dropwise to the aqueous solution of sodium lignosulfonate, and the mixture was allowed to react at room temperature for 30 min, ultimately yielding lignin-Ag.

### Preparation of hydrogel precursor solution

0.5 g of gelatin was dissolved in 5 mL of deionized water and stirred at 80 °C for 30 min to obtain a uniform gelatin aqueous solution. Subsequently, 1.5 mL of acrylic acid solution and 0.1 g of lignin-Ag were added, and the mixture was stirred at room temperature for 30 min. Then, 0.04 g of α-ketoglutaric acid was added, and the reaction continued for another 30 min to yield the hydrogel precursor solution (AGL).

### Preparation of S-FSR@AG and S-FSR@AGL patch

A certain amount of hydrogel precursor liquid was uniformly added to the hydroxylated surface of S-FSR, irradiated with UV for 1 h, and air dried at room temperature to obtain the S-FSR@AGL patch. The S-FSR@AG patch was obtained by polymerizing a hydrogel precursor solution that does not contain lignin-Ag.

### Characterization

The cross-sectional morphology of the patch was obtained by scanning electron microscopy (SEM, ZEISS Gemini SEM 300, Germany). The infrared absorption spectrum of the sample was obtained through Fourier transform infrared spectroscopy (FTIR, Thermo Fisher Scientific Nicolet iS20, USA). The hydrophobic properties of the sample were obtained using a contact angle/surface tension meter (Kunshan Shengding SDC 350KS). The mechanical properties and adhesion strength of the sample were obtained using a universal mechanical testing machine (CMT6104, SANS Test Machine Inc., China).

### *In vitro* antibacterial experiments

The material (S-FSR@AG and S-FSR@AGL) were mixed with Methicillin-Resistant Staphylococcus aureus (MRSA, CCTCC 16465) or Escherichia coli (*E. coli*, ATCC 8099) (10^6^ CFU/mL), and then placed in a 37 °C incubator for a 12 h cultivation period. During this time, 10 μL of bacterial suspension was taken every hour for drop plating, and photographs were taken after the 12 h cultivation was complete. Every hour during this period, 100 μL of the bacterial suspension was taken and placed into a 96-well plate, and the OD value of the solution at 610 nm was recorded using a microplate reader.

The S-FSR@AGL patch were incubated with the bacteria for a duration of 12 h. Following this treatment, the bacteria were fixed in a 2.5% glutaraldehyde solution for 4 h, subsequently rinsed twice with PBS (Phosphate buffered saline), and then subjected to a series of graded alcohol dehydration steps (30, 50, 70, 90, and 100%, v/v) for 15 min in each concentration. Ultimately, the morphology of the bacteria was examined using SEM.

### Anti-fecal and bacterial adhesion experiments

Two 0.1 g samples of mouse feces were weighed out, and an equal amount of water was added to each, followed by thorough mixing to create a uniform suspension. The wet mouse feces were then evenly applied to the surfaces of SR and FSR. Afterward, the same volume of water was poured from the same height onto the material surfaces to flush away the feces. Finally, photographs were taken to document the amount of feces on the material surfaces before and after the washing process.

SR and FSR were placed at the bottom of a 96-well plate, and 200 μL of MRSA or *E. coli* suspension (10^9^ CFU/mL) was added. The plates were incubated in a 37 °C incubator for 12 h. Subsequently, the supernatant was removed, and the samples were gently rinsed three times with a PBS solution, followed by fixation with a 2.5% glutaraldehyde solution for 4 h. After being washed twice with PBS, the samples underwent dehydration with a gradient of ethanol solutions (30, 50, 70, 90, and 100%, v/v) for 15 min each. Ultimately, the adhesion of bacteria on the sample surfaces was observed using SEM.

### Mechanical and adhesion property testing

The material was cut into rectangular strips measuring 1 cm × 5 cm. The upper and lower ends of each strip were secured to the fixtures of a tensile testing machine. The testing speed was set at 100 mm/min until the specimen fractured. The force and displacement of the material were recorded to obtain the stress-strain curve.

Rectangular strips measuring 1 cm × 5 cm were cut from both the patch and fresh pork tissue. The hydrogel layer of the patch was then adhered to the fresh pork tissue. One end of the patch and one end of the pork tissue were each secured to the fixtures of a tensile testing machine. The testing speed was set at 100 mm/min to peel the adhesive tape from the substrate, and the force data during the peeling process were recorded. By controlling the fixture clamping direction perpendicular to the substrate plane, a 180° peel test result was obtained, while controlling the fixture clamping direction parallel to the substrate plane yielded a shear test result.

### *In vitro* cytocompatibility evaluation

The cell viability was evaluated using the CCK-8 assay. hMSCs (human mesenehymal stem cells, sourced from Tongji Hospital in Wuhan, China.) were seeded in a 96-well plate, and hydrogel extracts were added (obtained from patches with a size of 1.5 cm² used for soaking). The plate was then incubated in a 37 °C incubator for 24 and 72 h. After incubation, 10 μL of CCK-8 solution was added to each well and incubated at 37 °C for 2 h. The OD value of the solution at 450 nm was measured using a microplate reader. Cell survival rate (%) = [(A-C)/(B-C)] × 100%, where A is the OD value of the experimental group, B is the OD value of the control group, and C is the OD value of the blank well.

### Hemolysis assay

The rat blood was centrifuged at 4 °C (3000 rpm for 15 min) to obtain red blood cells after the removal of serum. The red blood cells were then washed three times with PBS and re-suspended in PBS to create a 10% red blood cell suspension. Next, 1 mL of the sample PBS solution was mixed with 1 mL of the 10% red blood cell suspension and incubated at 37 °C for 4 h. After centrifugation, the OD value of the supernatant was measured at 570 nm. Hemolysis rate (RHR%) = (A_sample_ - A_PBS_)/(A_water_ - A_PBS_). A is the OD value. Water serves as a positive control, and PBS serves as a negative control.

### Antioxidant activity test of the S-FSR@AGL patch

The antioxidant activity of the sample was assessed using the DPPH/ABTS assay. A small piece of the patch (1.5×1.5 cm) was placed into a 4 mL centrifuge tube, into which 2 mL of a 9.7 μmol/mL DPPH solution in ethanol was added. Following a 30 min incubation in the dark, the absorption spectrum of the supernatant between 400-800 nm was monitored using a plate reader, and photographs were taken to document the color changes of the solution before and after incubation. ABTS**.**^+^ was generated by mixing ABTS with K_2_S_2_O_8_ in PBS and allowing it to stand in a dark environment at 25 °C for 4 h. Then, 2 mL of the ABTS**.**^+^ solution was added to the centrifuge tube containing the patch, and after 30 min incubation, the absorption spectrum of the supernatant between 400-800 nm was monitored using a plate reader, with photographs taken to document the color changes of the solution before and after incubation.

### Rat model of wound repair after hemorrhoid surgery

The animal experiment protocol in this study had been reviewed by the Laboratory Animal Management and Use Committee of the Second Affiliated Hospital of Chongqing Medical University, and it complied with animal protection, animal welfare, and ethical principles, in accordance with the relevant national regulations on laboratory animal ethics (IACUC-SAHCQMU-2024-00078). Rats weighing 200-220 g at 6-8 weeks were used for anal wound modeling. After successful anesthesia, the rectal mucosa was marked with methyleneblue at the incision site intended for modeling, with an incision diameter of 6 mm. After successful marking, the rectal mucosa, submucosa, and muscular layer were cut along the marked edge. Subsequently, the S-FSR@AGL patch (λpre patch=2) was applied to the incision. The experimental period lasted for one week, during which the experimental group was treated with the patch on the third and fifth days post-wounding. The control group's wounds were not specially treated after modeling. The rats were euthanized on the seventh day to conduct pathological examinations: H&E (hematoxylin-eosin) staining, Masson staining, Gram staining).

For the hemostatic performance test of the S-FSR@AGL patch, after creating the models in two groups of rats, one group was left untreated, while the other group was treated with the patch. Subsequently, filter paper was placed at the anal orifice, and the blood absorbed by the filter paper in both groups was statistically analyzed.

For the wound contraction experiment of the S-FSR@AGL patch, after rats were adequately anesthetized with chloral hydrate, their back hair was removed using a razor and depilatory cream. Subsequently, a punch biopsy instrument was used to create circular skin wounds with a diameter of 8 mm. The S-FSR@AGL patch was then applied to the wound surface, and the wound contraction was observed, with photographs taken every minute.

### Transcriptome sequencing and analysis

Anal samples from rat perianal tissues underwent total RNA extraction, followed by polyadenylated mRNA enrichment using the NEBNext® Poly(A) mRNA Magnetic Isolation Module. Fragmented mRNA was reverse-transcribed into double-stranded cDNA, and sequencing libraries were constructed with the Illumina Stranded mRNA Prep Kit. Paired-end sequencing (2×150 bp) was performed on the Illumina NovaSeq 6000 platform (Illumina, San Diego, USA). Raw reads were quality-trimmed and adapter-filtered via fastp (v0.23.4). Cleaned reads were aligned to the rat reference genome (mRatBN7.2) using STAR (v2.7.10a) with default parameters. Gene-level quantification was performed via featureCounts (v2.0.3) based on the Ensembl annotation (release 105). Differential expression analysis was conducted using DESeq2 (v1.38.3) under thresholds of |log2FC| > 1 and adjusted p < 0.05. All data analyses were executed on the Majorbio Cloud Platform (https://cloud.majorbio.com).

### Immunohistochemistry studies

The anal tissue from rats was fixed with 4% paraformaldehyde, embedded in paraffin, and sectioned. After dewaxing and rehydration, antigen retrieval was performed using citrate buffer (pH 6.0) / Tris-EDTA (pH 9.0) under high-pressure heat treatment (95 °C, 20 min), followed by blocking nonspecific binding sites with 5% BSA. The sections were then incubated with primary antibodies against ACTA1 (Biodragon, BD-PT0097, 1:200), MYL1 (Biodragon, BD-PE5017, 1:100), SPINK5 (Biodragon, BD-PT2554, 1:200), TP63 (Biodragon, RM4632, 1:100), IL-1 (Proteintech, 26048-1-AP, 1:200) and TNF-a (Proteintech, 26405-1-AP, 1:200) overnight at 4 °C. Subsequently, the sections were incubated with biotin-conjugated anti-rabbit or anti-goat secondary antibodies for 1 h at room temperature. Following DAB chromogenic reaction and hematoxylin counterstaining, the optical density (OD) values of positively stained areas within the anal tissues were quantified using ImageJ software.

### Statistical analysis

In this study, all results were expressed as mean values ± standard deviation with n ≥ 3. Inter-group comparisons were conducted using one-way ANOVA in conjunction with Student's *t* test. A P-value of less than 0.05 was considered statistically significant for all analyses.

## Results and Discussions

### Preparation and characterization of the S-FSR@AGL

**Figure 2:**
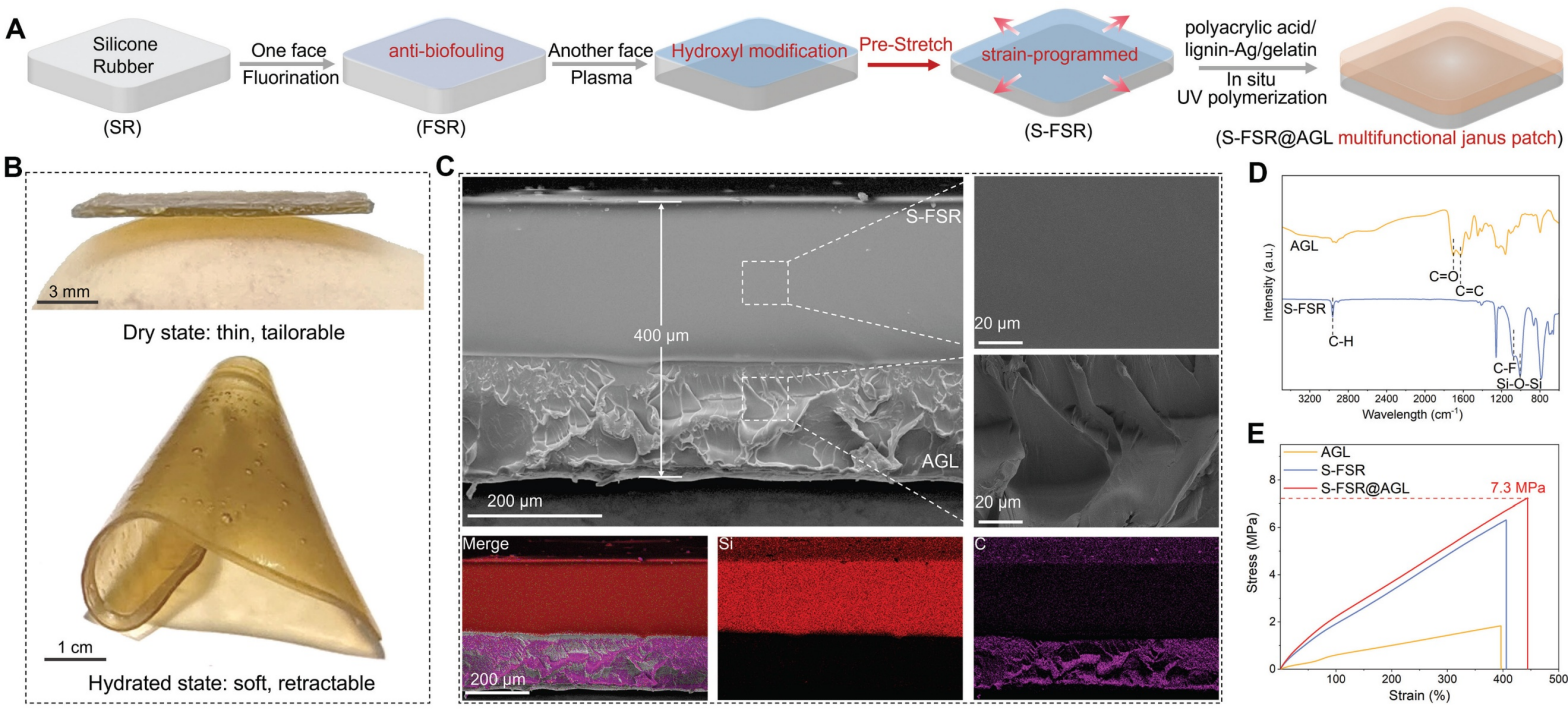
** Preparation and characterization of S-SFR@AGL patch.** A. Schematic diagram of the S-SFR@AGL fabrication process. B. Photographs of the S-SFR@AGL before and after moisturizing. C. SEM images of the S-SFR@AGL. D. FTIR spectra of the AGL and S-FSR. E. Tensile strength graph of the AGL, S-FSR and S-SFR@AGL.

Silicone rubber (SR) was selected as elastomer backing due to its great resilience and biocompatibility 17. Firstly, one side was fluorinated to prepare an anti-fouling rubber (FSR). Subsequently, the other side underwent plasma treatment to introduce a multitude of hydroxyl groups 18, which facilitates the combination of hydrogel consisted of acrylic acid/gelatin/lignin-Ag (AGL) on the surface. However, before the *in-situ* polymerization and drying of hydrogel, the fluorinated silicone rubber was pre-stretched (S-FSR) to induce a programmed strain to endow the patch with contractility (Figure 2A). As depicted in Figure 2B, the fabricated patch (S-FSR@AGL) was very thin, offering excellent tailorability and good ductility upon wetting. After wetting the patch, the silicone rubber and AGL did not peel off, indicating that the two layers were closely bonded. The peel test revealed that the peel force between the AGL hydrogel layer and the silicone rubber layer is 293.33 N/m, further indicating that the bond between the two layers is robust and secure. Besides, the patch was shrunk and crimped when it was wet, due to the soften of hydrogel and the retractive force of pre-stretched rubber. The thickness of patch was measured approximately 400 μm from the SEM image of cross-section. The silicone rubber layer was characterized by its dense structure, while the gel layer exhibits a loose and uneven texture. The two layers were seamlessly integrated along with separated distribution Si and C elements, demonstrating a strong bond between them (Figure 2C). In addition, the micro-morphology of the prepared lignin-Ag was observed. As shown in Figure S1, lignin-Ag consists of particles at the micrometer scale. The carboxyl groups (C=O, 1702 cm⁻¹) and benzene ring group (C=C, 1624 cm⁻¹) in AGL was confirmed in the FTIR spectra 19. In addition, a pronounced characteristic vibrational peaks at 1150 cm⁻¹ and 1005 cm⁻¹ were attributed to the C-F and Si-O-Si bonds, respectively, indicating the successful fluorination of rubber (Figure 2D) 20, 21. From the results of tensile test, AGL had a low tensile strength below 2 MPa due to the weak mechanical strength of polyacrylic acid hydrogel. However, after combining with S-FSR, the S-FSR@AGL patch had a high tensile strength of approximately 7 MPa and a maximum strain rate of around 440%, resulting from the high tensile strength of silicone rubber. The strong mechanical property of patch guaranteed its stability during everyday use in the complex environment of recta (Figure 2E). The ability to absorb water and swell is one of the key properties of hydrogels. To this end, the swelling behavior of AGL was tested in physiological saline at 37°C. As shown in Figure S2, upon immersing the dry AGL in physiological saline, the hydrogel immediately began to absorb water and swell. After 24 h, the swelling rate reached as high as 252%. This excellent water absorption capability ensures that when applied to a moist wound, the hydrogel can rapidly hydrate and adhere to the wound surface. During its application period, it can fully absorb the exudate from the wound, thereby maintaining the dryness of the wound.

### Anti-biofouling and antibacterial properties of the S-FSR@AGL

**Figure 3:**
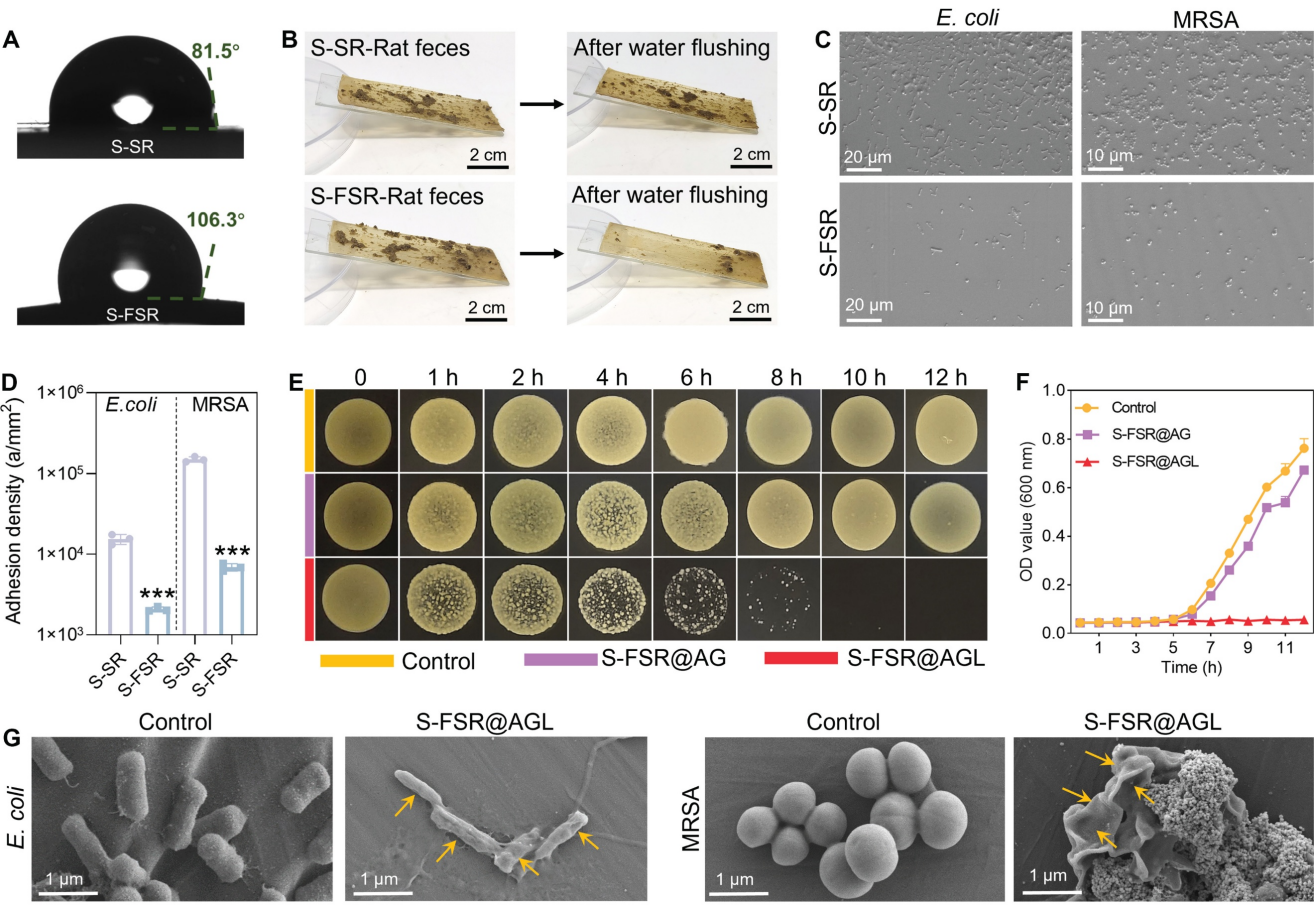
** Anti-biofouling and antibacterial effects of the S-FSR@AGL patch.** A. The contact angle of SR and S-FSR. Adhesion of rat feces (B), MRSA and *E. coli* (C) on the surfaces of SR and S-FSR. D. Quantitative statistics of MRSA and *E. coli* adhesion, n = 3 independent experiments per group, ***p < 0.001. Co-cultivation of MRSA with S-FSR@AG or S-FSR@AGL, with sampling at different time points for the drop plate images (E) and measurement of the bacterial suspension OD values (F). G. Morphologies of MRSA and *E. coli* after co-culturing with S-FSR@AGL for 12 h.

Since rectal are often exposed to feces and bacteria, it is essential to prevent the wound from their fouling. In accordance with our design philosophy, the non-adhesive side of the patch should possess superior anti-biofouling capabilities, while the adhesive side should maintain effective long-term antibacterial performance. As illustrated in Figure 3A, the contact angle of the pre-stretched silicone rubber (S-SR) was about 81.5°, while the contact angle of the pre-stretched silicone rubber after fluoride treatment (S-FSR) increased to 106.3°. Similarly, the rolling angle of pre-stretched silicone rubber (S-SR) was about 30°, and the rolling angle of the pre-stretched silicone rubber after fluoride treatment (S-FSR) decreased to 20° (Figure S3). This indicated that the hydrophobicity of the silicone rubber surface was enhanced after fluorination, which was conducive to its anti-biofouling performance 22. We placed an equal amount of moist rat feces to the surfaces of S-SR and S-FSR, rinsed with the same volume of water from the same height. The results showed that most of feces remained on the S-SR surface, while only a small amount was left on the S-FSR surface, demonstrating that S-FSR can effectively prevent fecal adhesion (Figure 3B). In addition, the adhesion of *E. coli* and MRSA to the surface of S-SR and S-FSR was also assessed. The bacteria were inoculated on the surface of the sample and rinsed with PBS after 12 h of culture. As shown in Figure 3C, compared to S-SR, there was a significant reduction in the number of bacteria adhering to the S-FSR surface. Further statistical analysis of the bacterial adhesion density revealed that the *E. coli* density on the S-SR surface was 15400 a/mm², and the MRSA density was 150333 a/mm². In contrast, the *E. coli* density on the S-FSR surface was 2078 a/mm², and the MRSA density was 20746 a/mm², indicating that S-FSR also can effectively prevent bacterial adhesion (Figure 3D). The above results demonstrated that the non-adhesive side of the patch (S-FSR) possessed an excellent anti-biofouling property. Subsequently, the antibacterial properties of the adhesive side of the patch were tested. As shown in Figure 3E, the bacteria were co-cultured with the patches, and the drop plates were sampled at different time points. The colony count in the S-FSR@AGL group progressively diminished with the duration of co-cultivation, ultimately reaching zero after 12 h. S-FSR@AG (without lignin-Ag) patch showed no antibacterial ability due to that the antibacterial effect only resulted from the constituent of lignin-Ag. At the same time, OD values of bacterial solution measured at different time points also showed the same trend (Figure 3F). Therefore, the ability of the patch to release silver ions was further investigated. As shown in Figure S4, the patch demonstrated a sustained release of silver ions over a period of three days, which endows the patch with good long-term antibacterial efficacy. The bacterial morphologies were further observed by SEM. Compared with the control group, both *E. coli* and MRSA in the S-FSR@AGL group were significantly wrinkled (Figure 3G). The above results demonstrated the excellent anti-biofouling and antibacterial properties of the S-FSR@AGL.

### Bioadhesive properties of the S-FSR@AGL

**Figure 4:**
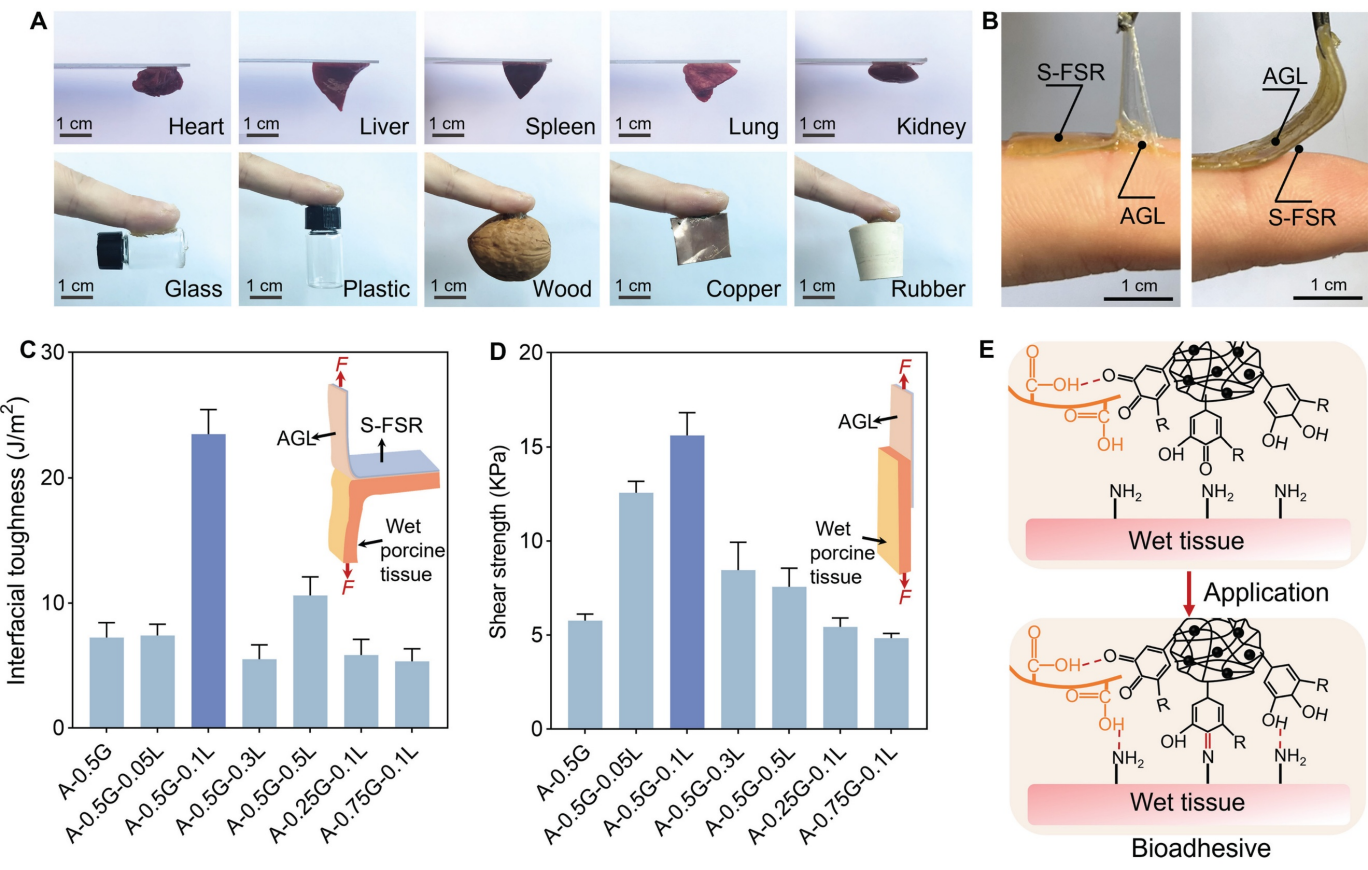
** Bioadhesive properties of the S-FSR@AGL patch.** A. Adhesion of the patch to various tissues and substrates. B. The adhesion of the patch's two sides to the tissue. C. Interfacial toughness (measured by ASTM F2256). D. Shear strength (measured by ASTM F2255) of the strain-programmed patch (λpre patch=2) and commercially available tissue adhesives on wet porcine skin, n = 3 independent experiments per group. E. Schematic diagram of the patch's adhesion mechanism to moist tissue.

The adhesive side (AGL) exhibited strong adhesion to various substrates, such as organs, glass, plastic, wood, metal, rubber (Figure 4A). We then tested the adhesion of both sides of the patch to tissue. As we designed, the S-FSR side did not exhibit adhesion to tissue, whereas the AGL side showed strong adhesion to tissue (Figure 4B). Subsequently, we used wet pork tissue as an adhesion model to test the interfacial toughness (by 180° peel test, ASTM F2256) and shear strength (by lap-shear test, ASTM F2255), thereby assessing the patch's adhesion performance to wet tissue 11, 23. By fine-tuning the mass ratio of acrylic acid (A), gelatin (G) and lignin-Ag (L) in the gel, the strain-programmed patch (*λ*pre patch = 2) of A-0.5G-0.1L could adhere firmly to the wet tissue after a light pressing for less than 5 seconds, with high interfacial toughness of more than 23 J m^-2^ (Figure 4C) and shear strength of more than 16 KPa (Figure 4D). These results indicated that the strain-programmed patch has a strong adhesive ability to wet tissue, which is due to the presence of carboxyl group in acrylic acid and catechol group in lignin-Ag NPs. The carboxyl and phenolic hydroxyl groups of the acrylic acid and lignin can form hydrogen bonds with amine of tissue 8, 24, 25. The catechol groups can establish covalent bonds with the tissue's amine or thiol groups through Schiff base formation or Michael addition reactions 25-27. These interaction endowed the patch with a strong affinity for wet tissue (Figure 4E).

### Controllable programmed strain and biocompatibility of the S-FSR@AGL

**Figure 5:**
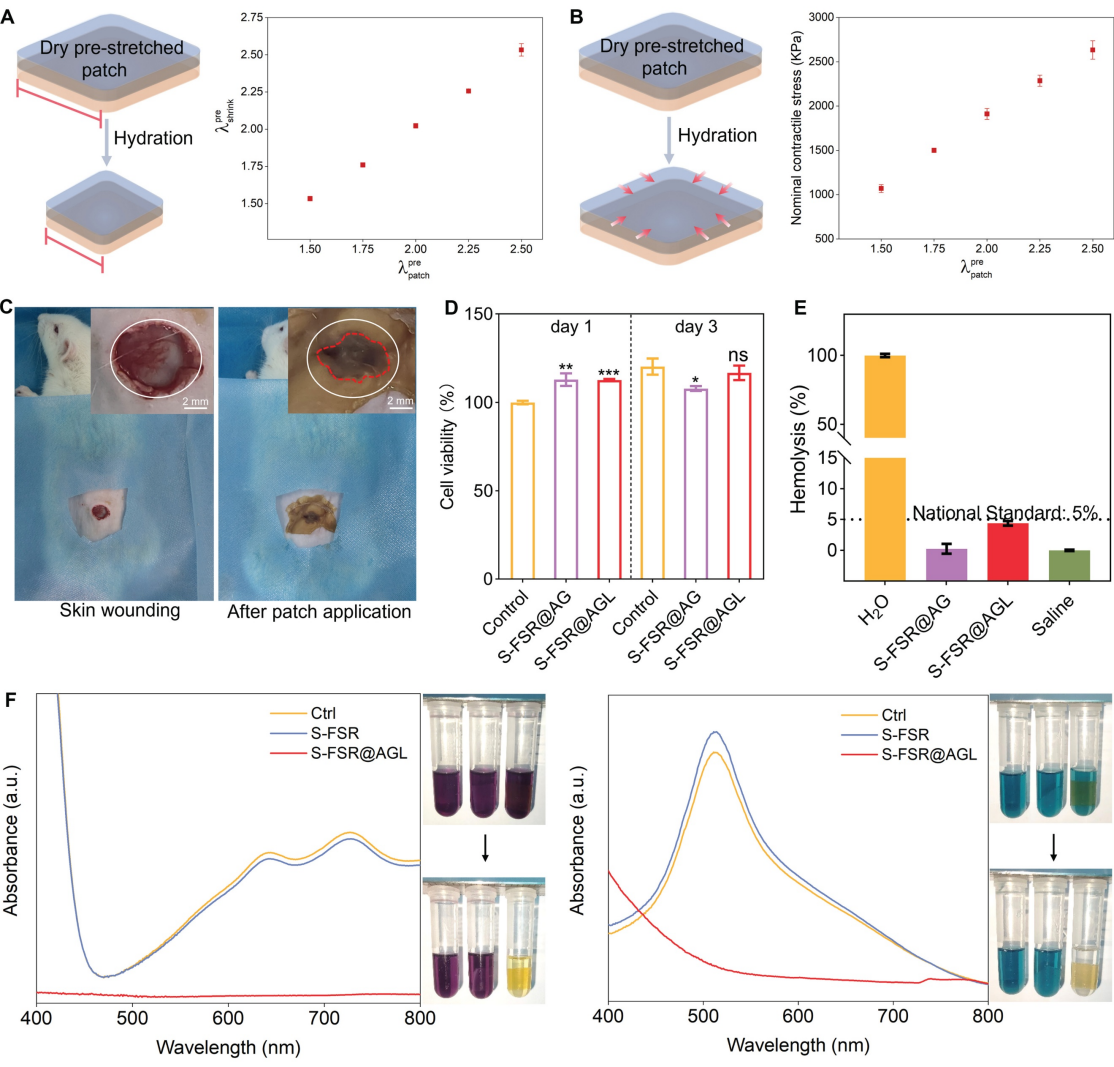
** Controllable programmed strain, biocompatibility and antioxidant capacity of the S-FSR@AGL patch.** A. Experimental values of contraction λpre shrink. B. Contractile stress generated by programmed strain release upon hydration of the strain-programmed patch with varying λpre patch, n = 3 independent experiments per group. C. Representative images of SD rat dorsal wounds before (left) and after (right) the S-FSR@AGL patch (λpre patch=2) application. D. The toxicity of S-FSR@AG and S-FSR@AGL to hMSCs, measured by CCK-8 assay, n = 3 independent experiments per group, *p < 0.05, **p < 0.01, ***p < 0.001. E. Hemolysis rate of S-FSR@AG and S-FSR@AGL toward red blood cells, n = 3 independent experiments per group. F. Antioxidant capacity testing of the S-FSR@AGL patch, DPPH (left) and ABST (right) assay.

The rubber side of patches was uniformly pre-stretched by a certain ratio (*λ*pre patch=pre-stretched length/original length), then the hydrogel side was dried into a glassy polymer to introduce programmed strain. Upon exposure to a moist environment, the hard hydrogel became soften after adsorbing water, inducing the uniform contraction of the strain-programmed patch (Figure 5A). Throughout this contraction phase, a contractile mechanical stress was generated, with a maximum reaching 2500 KPa, which can be applied to wound contraction (Figure 5B). It is worth noting that the relatively high mechanical stress generated by contraction gradually diminishes as the patch retracts. Additionally, the wound contraction process is relatively rapid, which ensures that the stored mechanical stress does not cause damage to the tissue. After that, we evaluated the patch's contract ability to wet wound using a dorsal wound model of SD rat. The wound began to gradually contract just 5 min after the application of the patch, and the contraction was complete after 10 min, with the area of the contracted wound reduced to 43% of its initial size (Figure 5C and Figure S5). This strong wound contract ability of patch was attributed to the release of programmed stress stored within the patch and the tight adhesion to wound.

The biocompatibility of the patches was evaluated using hMSCs. Figure 5D illustrates that, in comparison to the control group, the cell viability for S-FSR@AG and S-FSR@AGL on the first day were 113.0% and 112.8%, respectively. On the third day, the cell viability for S-FSR@AG and S-FSR@AGLwere 107.9% and 116.8%, respectively. Subsequently, the hemolytic activity of the patches was further assessed through a hemolysis assay (Figure 5E). Upon a 4 h incubation with red blood cells, the hemolysis rates for both S-FSR@AG and S-FSR@AGL were below the national standards (5%). These results proved that the patches possess excellent biocompatibility, confirming their biosafety 28. Considering the abundant catechol groups of lignin, the oxygen atoms on the phenolic hydroxyl groups can directly reduce free radicals by providing electrons. 29 The anti-oxidative property of S-FSR@AGL was measured using 2,2-diphenyl-1-picrylhydrazyl (DPPH) and 2, 2′-azinobis (3-ethylbenzothiazoline-6-sulfonic acid) diammonium salt (ABTS). The UV-vis absorption of DPPH and ABTS tests showed significant absorption peaks decline and color change after culturing with S-FSR@AGL, proving its strong ROS scavenging ability. In contrast, S-FSR alone exhibited no significant antioxidant activity (Figure 5F).

### *In vivo* treatments of rectal wound after hemorrhoids operation

**Figure 6:**
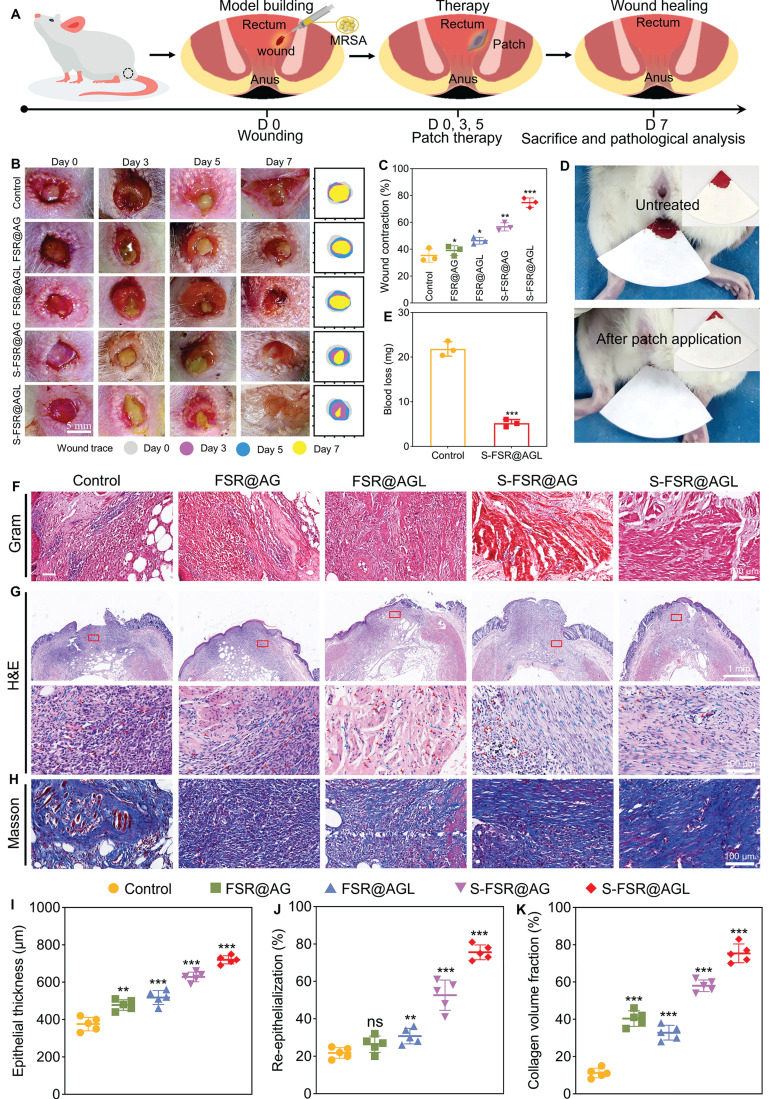
***In vivo* treatments of rectal wound after hemorrhoids operation.** A. Flowchart of the rat hemorrhoidectomy wound repair model. The dynamic morphological changes of the wound within seven days after treatment with different patches (B), and the statistical analysis of the wound contraction rate on the 7th day (C), n = 3 independent experiments per group, *p < 0.05, **p < 0.01, ***p < 0.001. The hemostatic effect of the patch on rectal wounds (D) and the statistical chart of bleeding volume in rats (E), n = 3 independent experiments per group, ***p < 0.001. Gram staining (F), H&E staining (G), and Masson staining (H) of rectal tissue on the 7th day after treatment with different patches. The results of epithelial thickness (I), re-epithelialization (J) statistically analyzed from H&E staining results, and collagen volume fraction (K) statistically analyzed from Masson staining results, n = 5 independent experiments per group, **p < 0.01, ***p < 0.001.

Subsequently, a rat model of mimicking the post-hemorrhoidectomy wound was established to validate the effectiveness of the patch. Following the creation of rectal wounds and adding MRSA on the wounds of rats, the patches were administered for treatment on the day 0, 3 and 5. The rats were euthanized on day 7 to conduct pathological examinations (Figure 6A). Rectal wound photographs were captured at various time points (day 0, 3, 5, and 7) to monitor the wound healing progress (Figure 6B). During the experimental process, the rats defecated normally every day. As shown in Figure S6, after two days of use in the rectal environment, the surface of the S-FSR@AGL patch remains clean and free of fecal contamination, indicating its excellent anti-fouling properties *in vivo*. In addition, it can be clearly seen that on day 3, there was still a residual patch on the wound (the yellow part), indicating that the patch has a strong adhesion to the wound and will not fall off due to the pressure of the rats' daily defecation. Moreover, the gelatin component within the hydrogel layer can be degraded by certain enzymes in the body, which subsequently leads to the disassembly of the entire hydrogel polymer network 12. However, during the animal experiments, the patch remained firmly in place on the wound site even after three days of application. This indicates that under normal dressing change frequencies, the patch will not prematurely fall off due to swelling or degradation. On day 5, the obvious fester was observed in control due to the infection induced by MRSA, while no fester was showed but some residue of patch in the wound of S-FSR@AGL group. On day 7, it can be seen that the wound was almost healed without swelling and exudate in the S-FSR@AGL group, which exhibited the best therapeutic effect compared to other groups. A statistical analysis of the bacterial content in the rectal wound tissue revealed that the bacterial count in the rectal tissue of rats treated with the S-FSR@AGL patch was the lowest among all groups, indicating that the patch possesses excellent *in vivo* antimicrobial properties (Figure S7). The wounds contraction was calculated from the wounds' area (Figure 6C). We found that all patches were helpful to the healing of wounds, among which, the S-FSR@AGL patch resulted in a maximum wound contraction (~80%). The great healing effect was attributed to, anti-biofouling, antioxidant, and wound adapted contracting ability of FSR@AGL patch. Since postoperative hemorrhage of hemorrhoids is very common, the hemostatic property of FSR@AGL patch was evaluated. Notably, after the excision of recta tissue, the use of the patch greatly reduced the amount of bleeding (Figure 6D-E). Subsequently, a detailed pathological analysis was performed on the tissues of the wounds. As shown in Figure 6F, Gram staining results revealed a large number of bacteria in the control group, while the bacterial count were markedly lower in the groups of FSR@AGL and S-FSR@AGL. The bacterial reduction was attributed to that the patches contained ligin-Ag could kill the bacteria and prevent the wound from the fouling of fecal matter, minimizing bacterial exposure 26, 30. Hematoxylin and eosin (H&E) staining indicated an abundance of inflammatory cells in the control group, a result of infection 31. Comparatively, the S-FSR@AGL group showed the least number of inflammatory cells. The inflammation relief was due to two primary factors: the patch's superior antibacterial property that combat infection, and its robust antioxidant activity to alleviate the oxidative stress environment of inflammatory tissue (Figure 5F) 26, 29. Furthermore, both the S-FSR@AG and S-FSR@AGL groups displayed increased number of fibroblasts, a phenomenon attributed to the release of programmed stress within the patches, which facilitated the tissue repair (Figure 6G) 9, 32. In line with these findings, the S-FSR@AGL group demonstrated a marked improvement in both re-epithelialization and the thickness of the epithelial layer when compared to other groups (Figure 6I-J). The results from Masson staining revealed the highest collagen deposition in the S-FSR@AGL group, indicating the best wound healing performance (Figure 6H and 6K). In summary, the designed rectal wound repair patch holds significant clinical importance. Upon application to the wound, the gel layer first absorbs the exudate from the wound, maintaining a dry environment around the wound. Secondly, the lignin silver contained in the gel layer exhibits excellent antibacterial properties, which can prevent wound infection. Moreover, the silicone layer has strong anti-biofouling properties, which can prevent the adhesion of feces and intestinal bacteria. The patch is designed to be changed every three days, effectively avoiding the accumulation of exudates.

**Figure 7:**
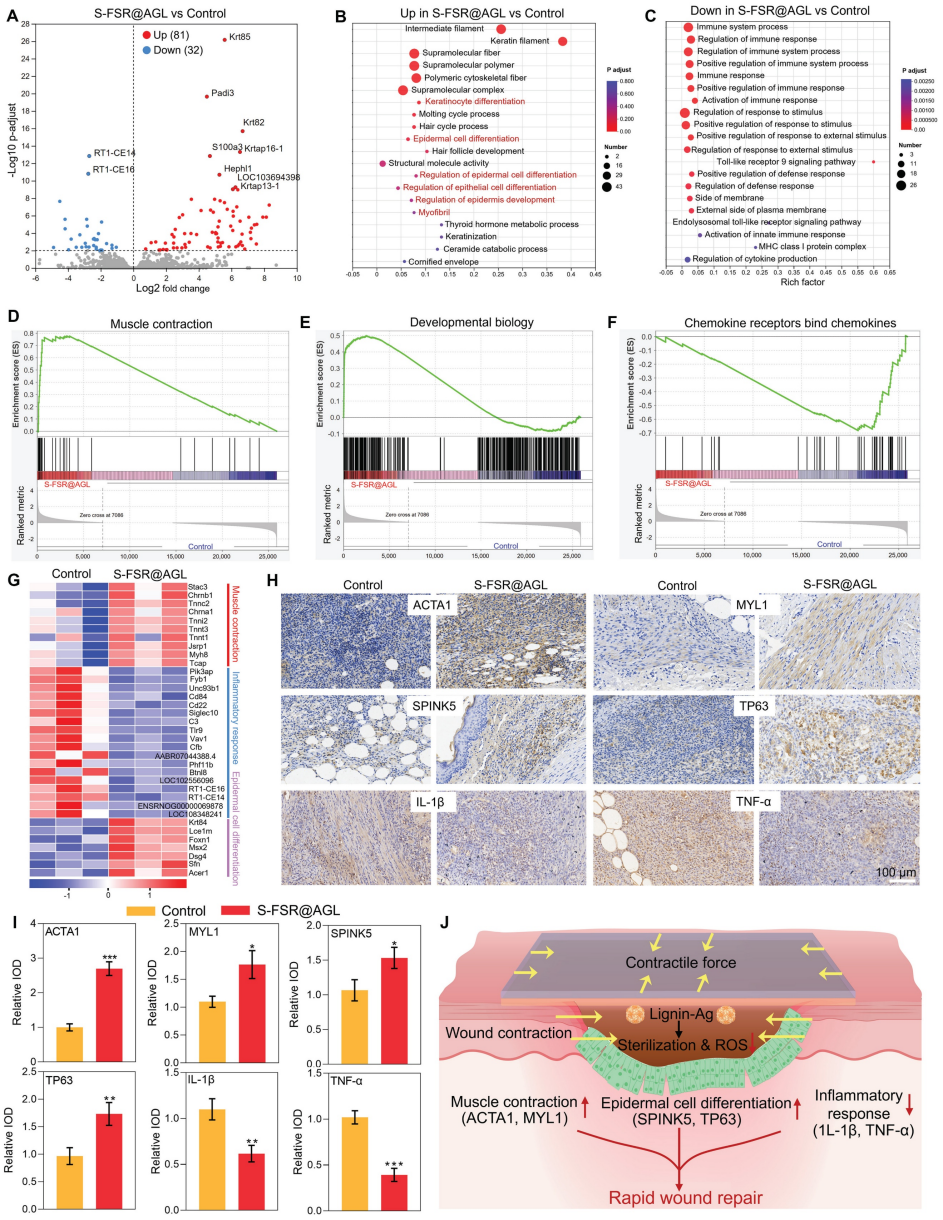
** Bioinformatic analysis.** A. Volcano plot of transcriptomic analysis of differentially expressed genes. n = 3 independent experiments per group. Upregulated (B) and downregulated (C) gene ontology (GO) enrichment analysis in S-FSR@AGL compared with Control. D-F. Gene Set Enrichment Analysis (GSEA) image. G. Heatmap analysis of differentially expressed genes involved in muscle contraction, epithelial cell differentiation and inflammatory responses. The range from -1 to +1 indicates the relative gene expression of S-FSR@AGL vs Control (log2 S-FSR@AGL/Control). Immunohistochemical staining of ACTA1, MYL1, SPINK5, TP63, IL-1β, and TNF-α in rectal tissue (H) and corresponding statistical results (I), n = 3 independent experiments per group, *p < 0.05, **p < 0.01, ***p < 0.001. (J) Therapeutic efficacy of the S-SFR@AGL patch.

To investigate the wound repair mechanism, a comprehensive transcriptomic analysis was conducted. As shown in Figure 7A, compared to the control group, there were 81 upregulated genes and 32 downregulated genes in the S-FSR@AGL group. Among the upregulated genes, the significantly changed genes (Krt85, Krt82, Krtap16-1, Krtap13-1, Padi3, S100a3, Hephl1, and LOC103694398) were associated with the proliferation and differentiation of epithelial cells. Conversely, among the downregulated genes, the significantly changed genes (RT1-CE14 and RT1-CE16) were linked to the inflammatory response. A Gene Ontology (GO) functional enrichment analysis was performed on these differentially expressed genes. As shown in Figures 7B-C, the upregulated genes were predominantly associated with tissue repair functions, including keratinocyte differentiation, epidermal and epithelial cell differentiation, while the downregulated genes were primarily linked to immune-related functions. Gene Set Enrichment Analysis (GSEA), presented in Figures 7D-F and Figure S8, revealed that in the S-FSR@AGL group, genes related to muscle contraction, developmental biology and keratinization were notably upregulated. In contrast, genes associated with chemokine receptor binding to chemokines were significantly downregulated. This suggested that the patch treatment significantly enhanced muscle contraction and epithelial cell differentiation while markedly reducing inflammatory responses, as evidenced by the gene expression heat map depicted in Figure 7G. Subsequently, to validate the transcriptomics, the immunohistochemistry was further conducted for analysis at the tissue level. The expression of ACTA1, MYL1, SPINK5, TP63, IL-1β, and TNF-α in rectal tissue was examined. Figures 7H-I demonstrate that in the patch-treated group, genes associated with muscle contraction (ACTA1 and MYL1) and epithelial cell differentiation (SPINK5 and TP63) were significantly upregulated. The expression of proinflammatory factors (IL-1β and TNF-α) were decreased, revealing that S-FSR@AGL mitigated tissue inflammation by eradicating bacterial infection and relieving oxidative stress. Simultaneously, it facilitated wound contraction by deploying programmed stress, thereby modulating the wound's mechanical microenvironment. This mechanical signal was conveyed to cells via the extracellular matrix (ECM) and extracellular fluid, initiating a cascade of signals that regulated tissue remodeling. This process encompassed the stimulation of epithelial cell proliferation and differentiation, collagen deposition, and muscle contraction, which further promoted active wound contraction 9, 33-35. Consequently, this multifunctional patch significantly accelerated the wound healing process of recta (Figure 7J).

## Conclusion

In summary, to address the clinical challenge of postoperative care of hemorrhoids, a lignin-based strain-programmed Janus patch (S-SFR@AGL) was developed. The ingenious design of the double sides ensured that the wound contact side delivers robust bioadhesion, antibacterial, antioxidant, and hemostatic functions, while the non-contact layer provides persistent anti-biofouling efficacy and generates programmable contractile force through pre-stored stress release. This dual functionality prevents fecal/bacterial contamination and actively contracts the wound by modulating the mechanical microenvironment. Validation experiments using *in vitro* and *in vivo* models have demonstrated that this lignin-based patch has beneficial therapeutic effects on wound healing. The transcriptomic analysis indicated that it could upregulate muscle contraction and epithelial cell differentiation at the wound site, as well as downregulate inflammatory responses to achieve rapid wound healing within a week. This easy-to-apply, multifunctional patch represents a clinically viable strategy for rapid rectal wound regeneration.

## Supplementary Material

Supplementary figures.
