# Supplementary material for: A strain-programmed lignin-based Janus patch for rapid healing of postoperative rectal wounds
Source: Theranostics. 2025 Jun 20;15(15):7425–39. doi: 10.7150/thno.115444 (PMC12315808; doi:10.7150/thno.115444)
Supplement: Supplementary file 1 — Supplementary figures. [file thnov15p7425s1.pdf]

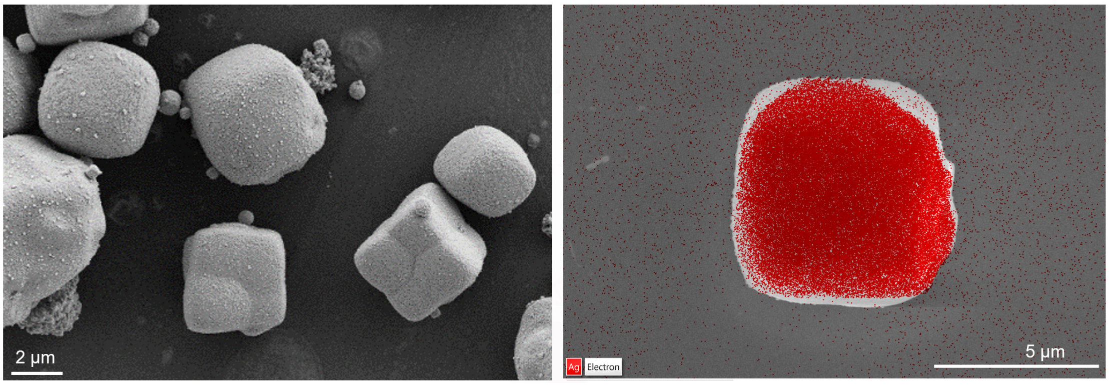

Figure S1. The SEM of lignin-Ag

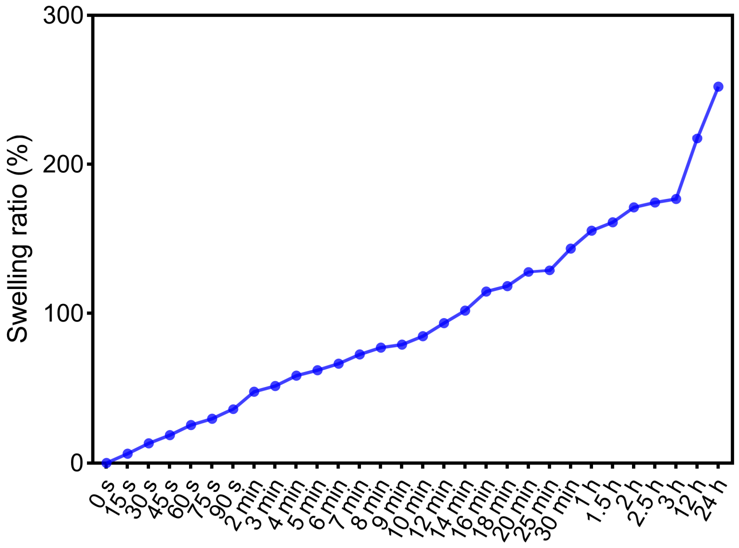

Figure S2. Swelling behavior of the hydrogel.

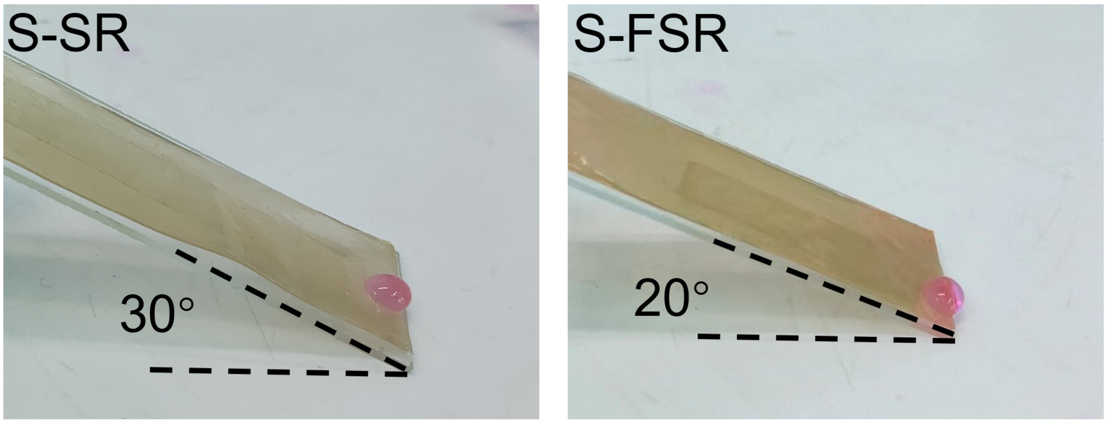

Figure S3. The rolling angle of S-SR and S-FSR

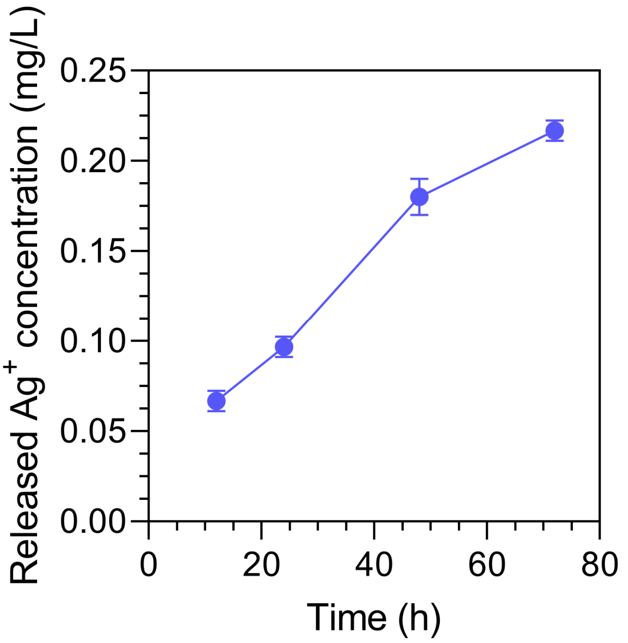

Figure S4. Cumulative Ag^{+} release curves of S-FSR@AGL patch at 37 °C in saline solution for long-term release (72 h).

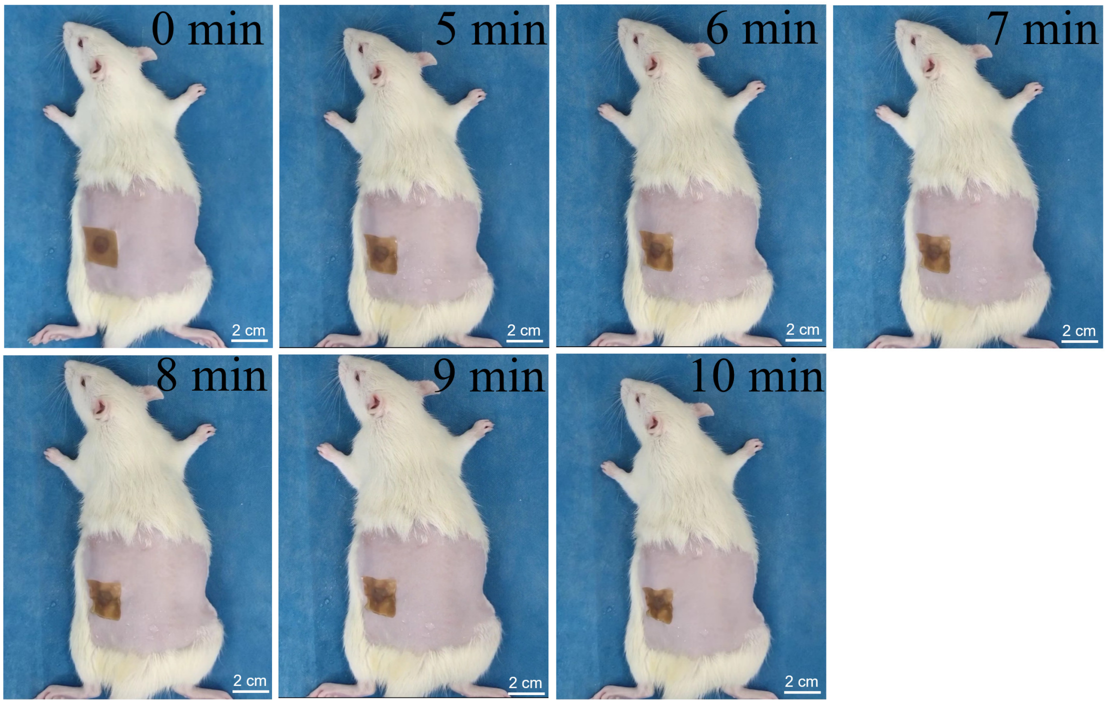

Figure S5. Representative images of SD rat dorsal wounds and after the application of S-FSR@AGL patch at different time points.

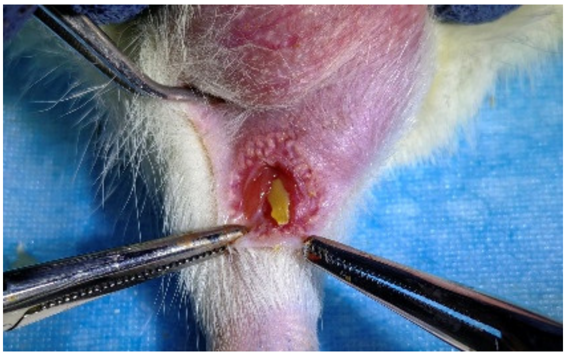

Figure S6. The actual photo of the patch after two days of application on the rectal wound.

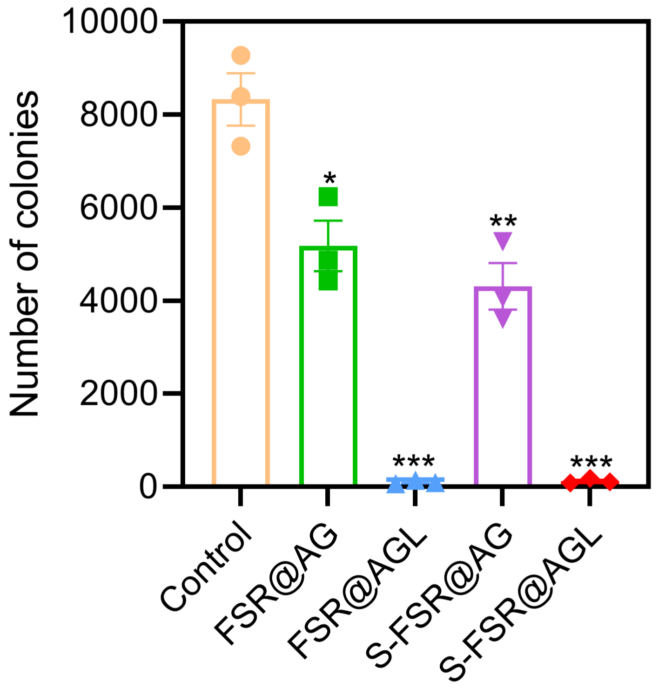

Figure S7. A statistical analysis of bacterial content in the rectal wound tissue of rats on the seventh day after treatment with different patches.

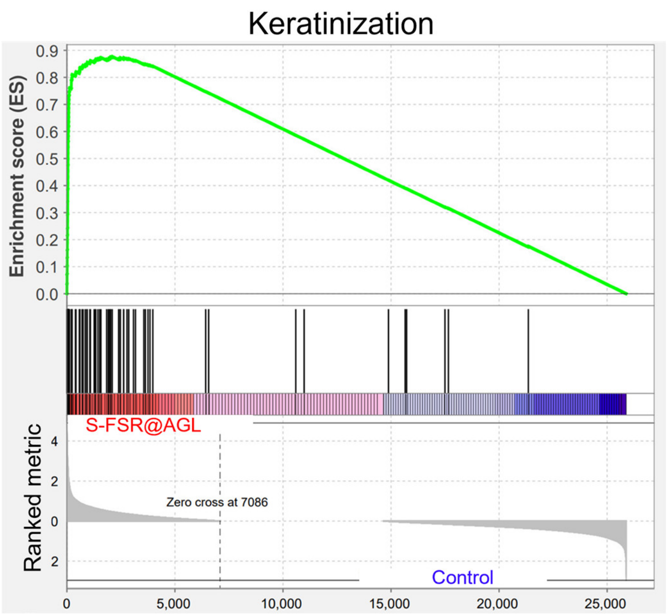

Figure S8. Gene Set Enrichment Analysis (GSEA) image.
